# Supplementary figures and images for: Unique features of the arterial blood–brain barrier
Source: Fluids Barriers CNS. 2023 Jun 27;20:51. doi: 10.1186/s12987-023-00450-3 (PMC10294539; doi:10.1186/s12987-023-00450-3)

Additional file 1: Figure S1

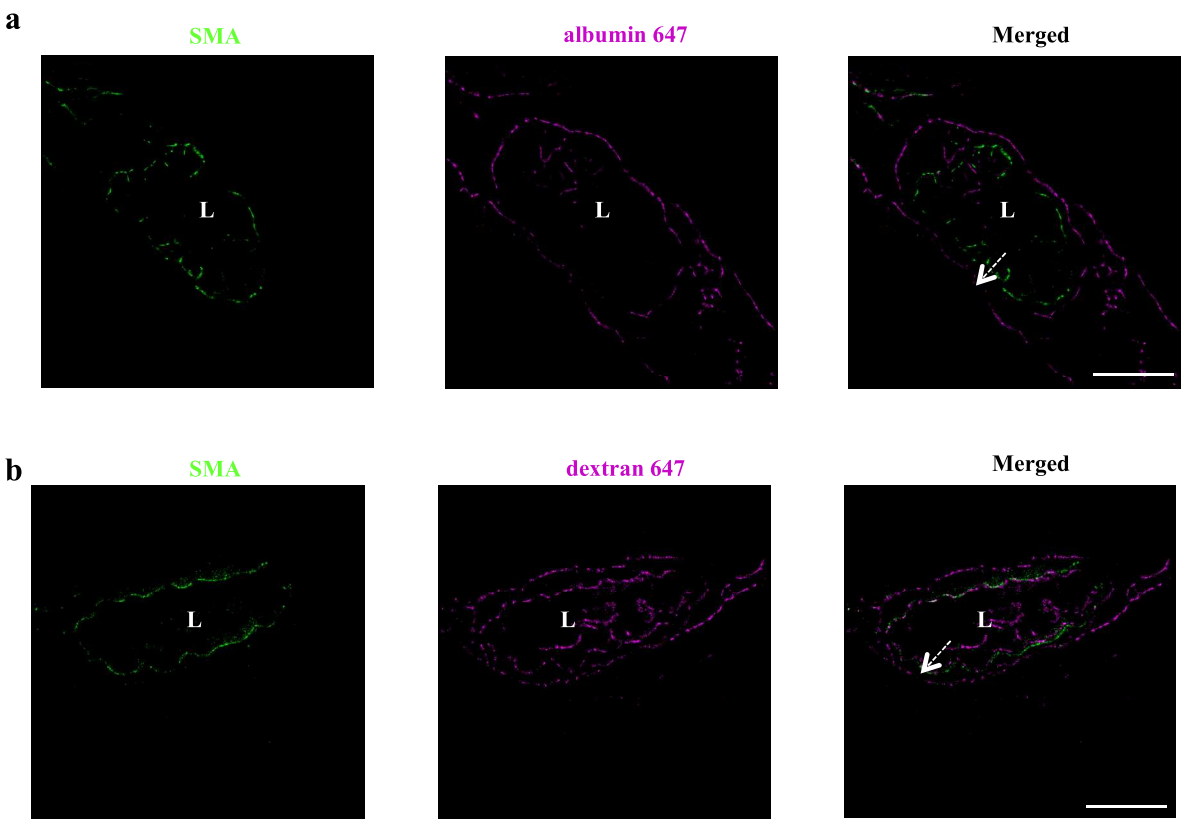

Additional file 1: Figure S2

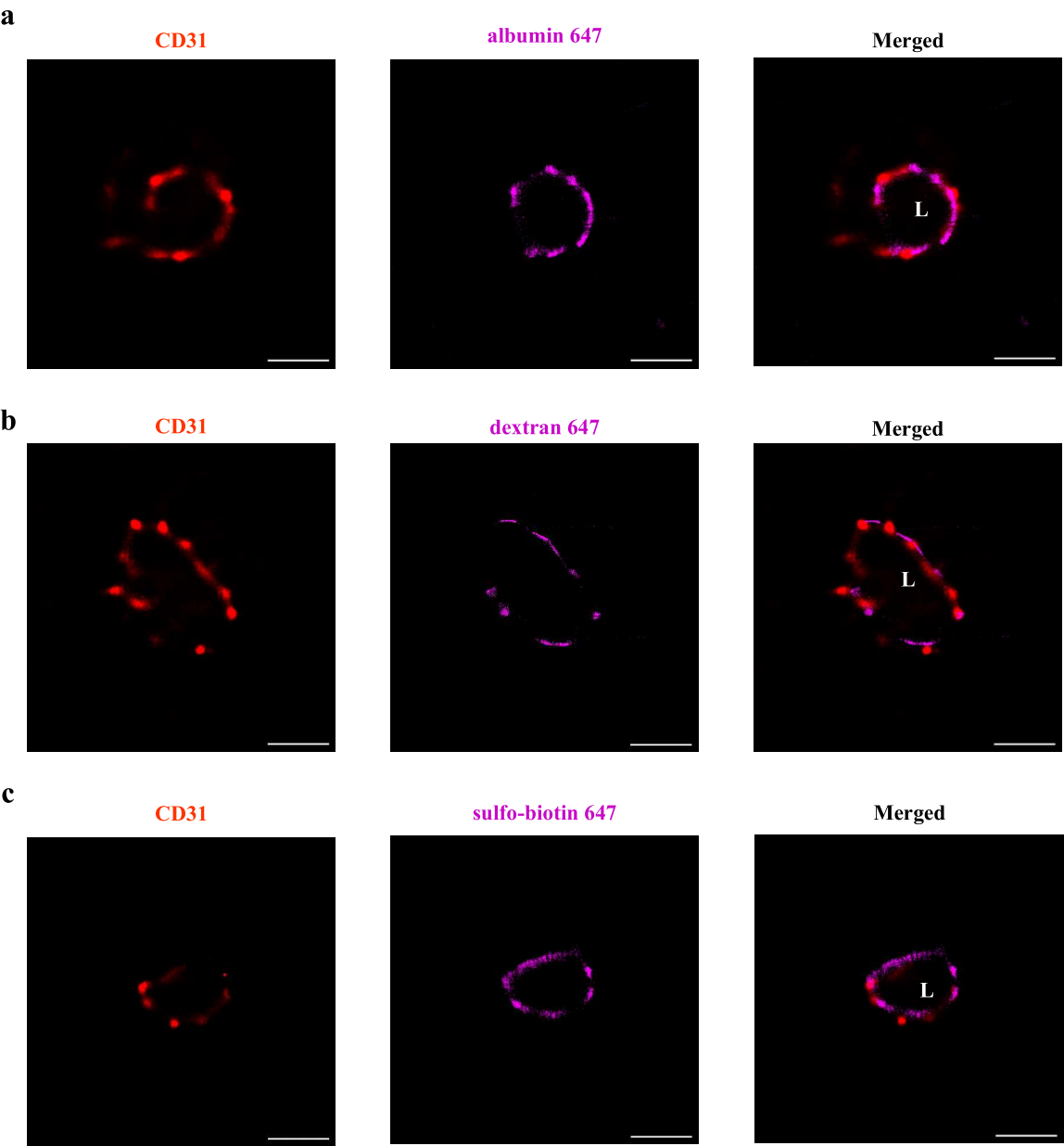

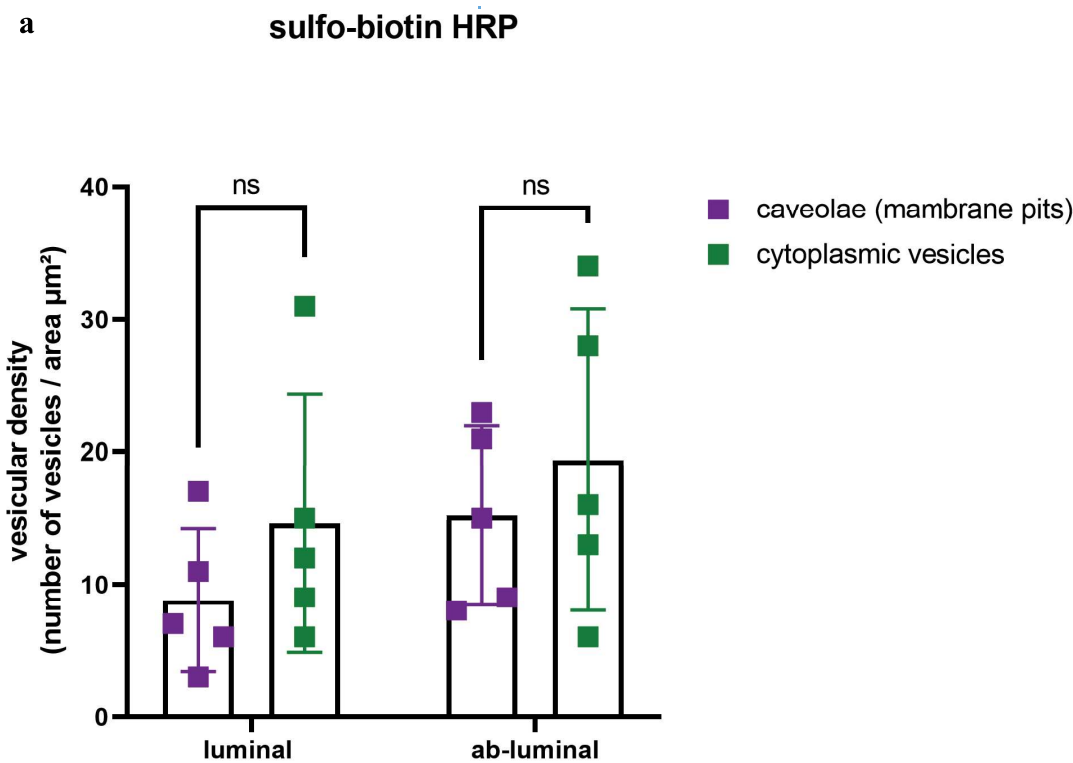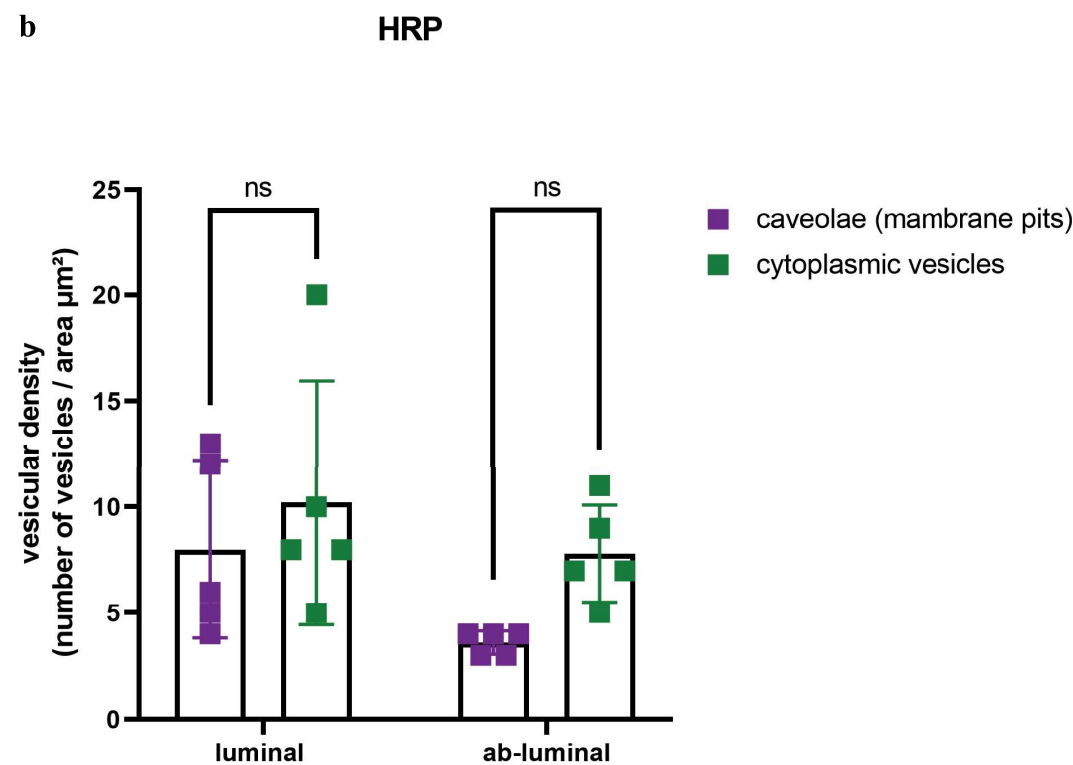

Supplement: Supplementary file 1 — Additional file 1: Figure S1. dSTORM imaging demonstrates super-resolution tracer permeability, validating unique barrier properties of the CNS arteriole-wall. Adult wild-type mouse cortical sections stained with SMA and imaged in dSTORM following tracer injections. Both albumin signals (a) and dextran signals (b) are found passed SMA markers. (L) marks the vessel lumen. Dashed arrows marks tracer direction from the lumen towards the parenchyma. Scale bar 10 µm (n = 6 mice, 24 arterioles). Quantification is shown in Fig. 2c. Figure S2. dSTORM imaging demonstrates super-resolution tracer permeability, confirming endothelial barrier properties of CNS capillaries. dSTORM imaging of wild-type adult mouse cortical capillaries, following tracer challenges from the blood (retro-orbital injections), (L) marks the lumen. Sections were stained for an endothelial marker (CD31). Tracer challenge demonstrates that all three tracers are mostly co-localized with CD31 (a, albumin 647 (70 kDa), b, dextan 647 (10 kDa), and c, sulfo-biotin 647 (443 Da)). Scale Bar 10 µm (n = 9 mice, 27 capillaries). Figure S3. Vesicle density in mouse arterial smooth muscle cells. Quantification comparing mean density of caveolae (membrane pits) and cytoplasmic free vesicles in areas adjacent to the luminal and ab-luminal membranes. Analysis corresponds to imaging presented in Fig. 3, of experiments with sulfo-biotin (a) or HRP (b) tracer challenges from the blood (retro-orbital injections). Data are mean ± s.e.m. *p < 0.05 (Two tailed Mann–Whitney U- test, n = 5 smooth muscle cells TEM profiles). [file 12987_2023_450_MOESM1_ESM.pdf]
